# Epigenetic Regulation of Circadian Clocks and Its Involvement in Drug Addiction

**DOI:** 10.3390/genes12081263

**Published:** 2021-08-19

**Authors:** Lamis Saad, Jean Zwiller, Andries Kalsbeek, Patrick Anglard

**Affiliations:** 1 Laboratoire de Neurosciences Cognitives et Adaptatives (LNCA), UMR 7364 CNRS, Université de Strasbourg, Neuropôle de Strasbourg, 67000 Strasbourg, France; lamisaad_8@hotmail.com (L.S.); zwiller@neuro-cnrs.unistra.fr (J.Z.); 2 The Netherlands Institute for Neuroscience (NIN), Royal Netherlands Academy of Arts and Sciences (KNAW), 1105 BA Amsterdam, The Netherlands; a.kalsbeek58@gmail.com; 3 Department of Endocrinology and Metabolism, Amsterdam University Medical Center, University of Amsterdam, 1105 AZ Amsterdam, The Netherlands; 4 Centre National de la Recherche Scientifique (CNRS), 75016 Paris, France; 5 Institut National de la Santé et de la Recherche Médicale (INSERM), 75013 Paris, France

**Keywords:** epigenetics, clock gene, drug addiction, DNA methylation, histone modification, neurodevelopment, histone deacetylase, substance use disorders, gene expression

## Abstract

Based on studies describing an increased prevalence of addictive behaviours in several rare sleep disorders and shift workers, a relationship between circadian rhythms and addiction has been hinted for more than a decade. Although circadian rhythm alterations and molecular mechanisms associated with neuropsychiatric conditions are an area of active investigation, success is limited so far, and further investigations are required. Thus, even though compelling evidence connects the circadian clock to addictive behaviour and vice-versa, yet the functional mechanism behind this interaction remains largely unknown. At the molecular level, multiple mechanisms have been proposed to link the circadian timing system to addiction. The molecular mechanism of the circadian clock consists of a transcriptional/translational feedback system, with several regulatory loops, that are also intricately regulated at the epigenetic level. Interestingly, the epigenetic landscape shows profound changes in the addictive brain, with significant alterations in histone modification, DNA methylation, and small regulatory RNAs. The combination of these two observations raises the possibility that epigenetic regulation is a common plot linking the circadian clocks with addiction, though very little evidence has been reported to date. This review provides an elaborate overview of the circadian system and its involvement in addiction, and we hypothesise a possible connection at the epigenetic level that could further link them. Therefore, we think this review may further improve our understanding of the etiology or/and pathology of psychiatric disorders related to drug addiction.

## 1. The Basics of Epigenetics

Epigenetics refers to the study of reversible, heritable changes in gene expression that do not involve changes in the DNA sequence [1]. Epigenetic mechanisms include histone modifications, DNA methylation and induction of non-coding RNAs [2,3]. Traditionally, euchromatin containing active DNA, which is open and amenable to transcription, is distinguished from heterochromatin in which DNA is condensed with a compact DNA–protein structure that cannot be transcribed. Histone modifications constitute a major part of epigenetic regulation, with the N-terminal tails of histones containing many residues prone to be modified. These modifications are considered to be highly dynamic, as many enzymes are required to create these epigenetic marks or to remove them. Each regulatory step can lead to different consequences for gene expression [4,5], with histone acetylation mainly resulting in increased transcription, whereas histone methylation can be associated with either transcriptional repression or activation. Moreover, not all these remodelling actions are independent, as a given modification may influence others and thus vary according to the gene considered [5,6,7].

Histone acetylation is an epigenetic modification characterised by the addition of an acetyl group on a lysine residue in histone proteins, by histone acetyltransferases (HAT). It leads to a more relaxed and less condensed chromatin state. Therefore, it is explicitly associated with increasing the propensity for gene transcription. Increments in histone acetylation generally favour learning and memory. Histone acetylation also promptly responds to neuronal activity in terms of neuronal depolarisation and synaptic plasticity [8,9]. Histone deacetylation is the reverse reaction to acetylation, where an acetyl group is removed. It is catalysed by histone deacetylases (HDACs), which play an essential role in gene regulation, in part because the DNA becomes more tightly wrapped leading to reduced access to transcription factors and limited efficiency of RNA polymerase II elongation. This leads to decreased gene expression levels, known as gene silencing [10].

Histone methylation is a process by which one, two, or three methyl groups are transferred to lysine and arginine residues of histones by histone methyltransferases (HMTs). The effects of histone methylation differ from one residue to another, as it was found to be associated with actively transcribed genes (H3K4me3), but also with repressed genes (H3K9me3 and H3K27me3) [7,11]. Histone methylation is a very dynamic process, occurring on various basic residues, and can either increase or decrease transcription of genes depending on the degree of methylation and the location of the methylated residue, and thus can provide different functional and phenotypic outcomes. It has also been associated with stimulating or inhibiting neural pathways related to the formation of long-term memories and learning, ageing, and neurodegenerative diseases [12,13]. In parallel, several classes of histone demethylases (HDM) reverse the action of the HMTs [6]. HDMs serve as important targets for several systems involved in addiction [14,15] and circadian rhythms [16]. 

DNA methylation is a relatively solid epigenetic process characterised by the covalent addition of a methyl group to the DNA base cytosine to form 5-methylcytosine (5-mC). The reaction is catalysed by DNA methyltransferases (DNMTs) and occurs predominantly at CpG dinucleotides, but can also occur in a non-CpG site. In mammals, three DNMTs play a pivotal role, DNMT1, DNMT3a and 3b that are respectively involved in maintenance and de novo DNA methylation. Note that these enzymes have overlapping and different target genes and functions [17,18]. Similarly, Tet proteins also display tissue-specific biological functions and recognise common and specific 5mC target sequences [19,20,21]. DNMT1 being considered as a maintenance enzyme, while DNMT3a and -b are considered as de novo DNMTs. DNA methylation occurring in a promoter region usually causes gene repression. The first step in DNA demethylation occurs by the removal of 5-mC via the sequential modification of cytosine bases by several enzymes and progressive oxidation. DNA demethylation consists first of the addition of a hydroxyl group to 5-mC to form hydroxymethylcytosine (5-hmC). The reaction is mediated by the ten-eleven translocation (TET) family (TET1, TET2, and TET3), enzymes that are abundantly present in the brain. These proteins bind to CpG rich regions to prevent unwanted DNMT activity. They catalyse the oxidation of 5-mC to 5-hmC and successive other oxidative products, such as 5-formylcytosine and 5-carboxylcytosine through hydroxylase activity [22]. Interestingly, 5hmC levels were found to display the highest levels in neurons [7,23,24,25,26].

While 5-methylcytosine generated by Dnmts has been considered as a fifth nucleotide in mammalian genomes [27], the discovery of its oxidative products (5-hydroxymethylcytosine, 5-formylcytosine and 5-carboxylcytosine) [22,24] has shown that this covalent DNA methylation modification was not as stable as initially thought. Indeed, this process is reversible through mechanisms involving ten-eleven translocation (Tets) methylcytosine dioxygenases and the base excision repair pathway [3,28]. Nevertheless, the role and function of these oxidative products as “potential additional nucleotides” together with that of their binding proteins in transcription and chromatin remodelling requires further investigation.

Together with histone modifications and DNA methylation, microRNAs (miRs) can be considered to play an epigenetic regulatory role. MiRs are a class of small, highly conserved endogenous non-coding pieces of RNA (ncRNA) of 21–25 nucleotides in length. They function in the post-transcriptional regulation of gene expression, by interacting with various mRNAs through complementary base-pairing to influence the translation or stability of their target mRNAs [29,30]. The regulations between miRNAs and target genes are highly time and tissue specific. Moreover, recently, miRNAs have been considered as epigenetic modulators, forming a miRNA-epigenetic feedback loop that extensively impact on gene expression proliferation [31,32,33].

## 2. The Circadian Timing System

The circadian clock is an endogenous, self-sustaining pacemaker, found in most living organisms. It operates with a periodicity of ~24 h, to maintain daily rhythms for many metabolic processes, physiological functions, and behaviours, i.e., sleep–wake cycles, neuronal and cognitive functions, glucose metabolism, body temperature, and hormone secretion [34,35,36,37], in the absence of environmental inputs [38,39,40]. Those rhythms can be adjusted by factors called zeitgebers, with light being the dominant zeitgeber, but also including cues such as temperature, diet, exercise, and gravity. This adjustment is crucial for keeping the ~24 h rhythm synchronised with the exact 24 h day/night rhythm of the earth, and anticipating recurrent daily and seasonal changes in the environment [41,42]. In mammals, the suprachiasmatic nucleus (SCN), a small brain region in the anterior hypothalamus, operates as a focal regulator of circadian rhythms throughout the rest of the brain and body [43,44]. Phase resetting of the clock neurons in the SCN is accomplished via the light-induced release of glutamate from retinal projections that reach the hypothalamus via the retino-hypothalamic tract [45]. The circadian timing system provides both temporal synchrony for a range of cellular processes, as well as a molecular regulatory process that modulates gene expression at virtually all possible levels and has clear and immediate effects on behaviour [46,47]. Many so-called clock-controlled genes (CCGs) have been shown to be expressed in a daily rhythm, i.e., approximately 10% of transcripts in the genome show daily oscillations in a tissue-specific manner [48,49]. Yet, only a small set of genes are part of the molecular clock mechanism itself and usually are referred to as the core or classic clock genes (CGs). These include Clock (CLK), Brain and Muscle ARNT-like 1 (BMAL1), Period (PER), Cryptochrome (CRY), reverse strand of ERBA (REV-ERBα), retinoic acid related-orphan receptor alpha (RORα), and a few others [41,50]. Most of these CGs encode proteins that act as transcription factors to initiate the rhythmic expression of their target genes, with some of them forming heterodimer complexes [51,52], incorporating a transcriptional-translational autoregulatory complex such as CLK-BMAL1, and PER-CRY [41].

## 3. Epigenetics, Circadian Timing and Addiction

An electronic search was performed with online published papers, using the following keywords: Drugs of abuse, neuroepigenetics, DNA methylation, Dnmt, Tet, MBD, nutrition, diet, circadian rhythms, addiction, compulsive behavior/disorder, core-clock genes, appetite and satiety, natural reinforcers, reward, chromatin remodeling, and histone code. This search was performed within the PubMed (https://pubmed.ncbi.nlm.nih.gov/ (accessed on 1 November 2020)), Cochrane Library (https://france.cochrane.org/cochrane-library (accessed on 1 February 2021)) and Web of Science online databases. Articles were selected based on citation index, journal impact factors (https://www.bioxbio.com/ (accessed on 1 March 2021)), quality of technical methods, and personal appreciation. Other databases have been consulted for gene and protein sequences at https://www.ncbi.nlm.nih.gov/ (accessed on 1 January 2021), as well as the microRNA database https://www.mirbase.org/ (accessed on 1 April 2021) or genome-wide analyses associated with published papers.

## 4. Epigenetics in Circadian System

Beyond the classic core clock genes, the regulation of circadian transcription is modulated by many other genetic and epigenetic factors. Thus, the core clock feedback loop is insufficient to explain all the observations, especially those relating to human behavioural traits and disorders [53,54]. Moreover, epigenetic mechanisms are crucial mediators of environmental factors that modulate rhythmic gene expression. These vast changes in the epigenetic state alter dynamically over the day-night cycle [41]. Circadian transcription and rhythmic chromatin modifications together regulate oscillations in gene expression. Rhythmic histone acetylation (H3K9, H3K14) was demonstrated on the promoter regions of CCGs [53,54,55], connecting histone acetyltransferase (HAT) p300 [56] and the intrinsic CLOCK HAT activity [55,57]. The latter was counteracted by the NAD(+)-dependent deacetylase sirtuin 1 (SIRT1) [58,59] that adjusts the circadian acetylation of histones and non-histones [60].

Histone acetylation/deacetylation is the epigenetic mechanism that CLK uses to regulate circadian rhythms. The HAT p300 together with the Clock/Bmal1 complex regulates histone H3 acetylation at the *Cry* and *Per* promoters to influence their expression [56]. Furthermore, CLK itself possesses HAT activity by which acetylated BMAL1 recruits CRY1 to the CLK-BMAL1 complex and represses transcription [55,57]. Rhythms in acetylation of histone H3 in the *mPer1*, *mPer2*, and *Cry1* promoters were recorded peaking during the transcriptionally active phase [56,61]. Histone deacetylases (HDACs), like HATs, are important regulators of circadian rhythms and addiction-related phenomena [6], memory formation, as well as metabolism [57,62]. The HDAC3 subtype, which was found to be recruited by the nuclear receptor corepressor 1 (Ncor1), is involved in repressing *Bmal1* expression, thus affecting circadian rhythms. HDAC3 recruitment fluctuates, in conjunction with Reverb-alpha and Ncor, forming a HDAC3/Reverb-alpha/Ncor complex. The transcription of many genes oscillates in concert with either the fluctuation of HDAC3-related histone modification or with the complex-related signalling pathways [63,64]. On the other hand, HDAC inhibitors increase H3 acetylation and affect Per2 expression [62]. SIRT1, an NAD^+^-dependent histone deacetylase, interacts directly with clock genes by binding to CLK-BMAL1, promoting deacetylation and degradation of the PER2 protein in mice [58]. It is also a metabolic sensor, requiring NAD^+^ binding for its enzymatic activity, therefore linking the metabolic state to the circadian system. Besides, SIRT1 has been implicated in brain functions like ageing, neurodegeneration [65,66], synaptic plasticity, and memory formation [67,68].

Methylation and demethylation of histones also modify circadian-regulated gene expression [54,69,70]. Rhythmic histone methylation at H3K4, H3K9, H3K27, and H3K36 is catalysed at these spots by several HMTs and HDMs [53,70,71]. SUV39 methyltransferase, a critical regulator of rhythmic H3K9 di-methylation (H3K9me2), recruits CLOCK:BMAL1 to the E-boxes of CCG promoters [54,69,70,72]. Presumably, through the association of Suv39h with PER2, rhythmic discretional heterochromatin is controlled by H3K9me2 HP1 binding at DBP, PER1, and PER2 during the repressive phase [54,70]. EZH2 methyltransferase contributes to histone methylation, di- and tri-methylation of H3K27 (H3K27me2 and H3K27me3), and circadian gene expression of *mPer1* and *mPer2* [71]. The lysine-specific demethylase (LSD1), JumonjiC, and ARID domain-containing histone lysine demethylase 1a (Jarid1a) are major binding partners of CLOCK-BMAL1, thus enhancing transcription by CLOCK-BMAL1 [16]. LSD1 catalyses the removal of methyl groups from H3K4 and H3K9, associated with CLOCK and BMAL1. It has therefore been reported as a key component of the circadian machinery and regulator of CCG expression [73]. JARID1A has been implicated in circadian control by interacting directly with CLOCK:BMAL1 and regulating circadian gene expression. JARID1A also displayed demethylase-independent function not affecting the H3K4me3, but acting on and inhibiting HDAC1 recruitment. In contrast, overexpression of JARID1b and JARID1c can potentially reverse H3K4 methylation, but further studies are needed here [16]; also included in the mammalian circadian clock mechanism is histone demethylase JMJD5, which demethylates H3K36 and recruits several modifications, both activating or repressing ones, eventually inducing changes in the heterochromatin on a circadian timescale [74].

Furthermore, DNA methylation is a well-studied tissue-specific epigenetic modification considered to be important in gene regulation, generally by repressing gene transcription through recruitment of corepressor complexes (e.g., HDACs and HDMs) involving several DNA methyl-binding domain proteins (MBDs) [75,76,77]. Generally, it is considered as a rather stable epigenetic mark but it can also change with a 24 h cyclic pattern in genome-wide studies [78,79]. There is increasing evidence for crosstalk between dynamic DNA methylation and circadian rhythms, particularly in the central SCN clock [78,79,80], as for instance, shown by the light-induced changes in DNA methylation at specific promoters that correspond to circadian gene expression [79]. In human studies, this relationship between both systems has been evidenced by the 24 h variation in homocysteine levels and the global DNA methylation level [81,82,83]. Homocysteine levels have been linked to DNA methylation in many studies [84]. In parallel to that, an elevated homocysteine concentration correlates with drug addiction [85,86]. Moreover, sleep deprivation, and thus an altered circadian clock, can change the DNA methylation and hydroxymethylation of many CpG sites in genes involved in synaptic plasticity, signalling or neurotransmission, and others (for example, Arc, Egr1, and Neuroligin-1) [87,88]. An epigenome-wide study in mice demonstrated that an altered day-length changed gene expression profiles and patterns of DNA methylation in the SCN, suggesting that DNA methylation regulates the circadian clock in the SCN [79]. Another study in humans, using global statistics, showed that 24 h rhythmicity of DNA methylation correlated with rhythmic gene expression in the prefrontal cortex (PFC) [78,79]. In shift-workers, methylation changes are found in the *Clock* gene, being hypomethylated, while the *Cry2* gene was hypermethylated [88]. DNMT levels are expressed rhythmically in the mouse brain and liver, with some evidence of rhythmic DNA methylation occurring in Line-1 repeat elements [89,90]. Interestingly, the canonical E-box motif contains a central CpG moiety that is susceptible to become methylated, suggesting competition with BMAL1/CLOCK binding sites [91,92]. However, this has not been directly tested for BMAL1/CLOCK, so it has still to be determined whether this differential CpG methylation of E-boxes indeed regulates BMAL1/CLOCK genomic binding.

Finally, miRs, a genome-wide layer of circadian clock regulation [93], were found to impact the regulation of circadian rhythms, affecting gene expression and thus circadian output [94,95]. Dicer is the major enzyme in miR biogenesis. MiR-24, miR-29a [96], and miR-30a [97] have been reported to regulate circadian rhythms, specifically by targeting PER1 and PER2 and thereby determining the period of the cycle. Shorter circadian cycles due to faster translation of PER1 and PER2 proteins were found in Dicer-deficient cells and mice [98]. Rhythmic miR-132 and miR-212 are expressed in the central circadian clock and as they share the same seed region, they can target the same mRNAs. MiR-132/212 was found to exhibit a light regime-specific role in the circadian clock machinery, suggesting that they act as background-dependent circadian rhythm modulators [99]. MiR-212 and -132 also belong to an intronic polycistronic cluster activated by CREB and modulate dendritic plasticity by controlling MeCP2 expression [30,100]. MiR-134 and miR-132 are engaged in circadian regulation by targeting genes involved in chromatin remodelling (*Mecp2*, *Jarid1a* and *Sirt1*) that are implicated in *Per* and *clock* gene expression in the SCN [68,101]. Furthermore, both miR-124 and miR-181a expression are under circadian regulation and modulate circadian output by targeting Per3 as well as Cry1 [102]. Let7 modifies the circadian rhythm by regulating prothoracicotropic (PTTH), which is a direct target of CLOCK [103]. MiR-9 is involved in modulating circadian rhythms by targeting the *Clock* and *Sirt1* genes [104]. However, note that even if a microRNA and its complementary targets are present in the same cell, their possible interactions are still determined by their cellular localisation, underlining cellular or tissue specific interactions [31].

Finally, long non-coding RNAs (lncRNAs), too, have been shown to regulate circadian rhythms. Melatonin is an important hormone that times many seasonal rhythms. In the rat pineal gland, which is the source for the daily rhythm of melatonin in the circulation, a rhythmic expression has been described for lncRNAs [105,106].

## 5. Addiction and Epigenetics

According to the diagnostic and statistical manual of mental disorders (5th ed.) (DSM-5), the criteria of addiction are characterised by the presence of two or more of the following manifestations: recurrent continued use despite being aware of effect, tolerance, withdrawal syndrome, excessive use, persistent desire and others [107]. Drugs of abuse stimulate the brain reward circuitry, which concerns dopaminergic neurons originating in the ventral tegmental area (VTA) of the midbrain that project to the limbic system, in particular the nucleus accumbens (NAc), dorsal striatum, amygdala, hippocampus, and regions of the prefrontal cortex [108]. Psychostimulant drugs act by directly prolonging the effects of these dopaminergic signals. Thus, dopaminergic neurons located in several brain regions are activated by blocking the reuptake of dopamine (DA) into nerve terminals, thereby increasing DA levels in the synaptic cleft. The increased DA levels result in an augmented stimulation of various DA receptors located in brain circuits that subsume reward, mostly the D1- and D2-receptors [109]. One hypothesis states that the drugs induce long-lasting changes in the brain through a process known as homeostatic desensitisation. These alterations in gene regulation contribute to addictive behaviours. For instance, drugs alter the reward circuitry such that it causes increased motivation salience to drug cues, effectively making drug-related environmental stimuli more difficult to disregard and thus leading to intense drug craving and relapse [110]. Similar pathological alterations in other parts of this circuitry further impair behavioural control. Changes in the transcriptional potential of genes are established through the actions of transcription factors (FosB, NF-κB, CREB, MEF2), as well as chromatin remodelling (histone modification, DNA methylation) and noncoding RNAs, in particular miRs, which thereby contribute importantly to many of the observed neuroadaptations [6,111,112].

In response to drugs of abuse, the mesocorticolimbic dopaminergic activity in the VTA causes long-lasting plasticity in the glutamatergic neurons in the NAc and the PFC [108]. Chronic psychostimulant use affects the initial reliance on accumbens dopamine for drug reinforcement to reliance on the prefrontal and amygdala dopamine to trigger a relapse. For example, drugs act directly on NAc neurons that express opioid receptors, yet they promote dopamine release indirectly in the NAc by inhibiting gamma amino butyric acid (GABA)-ergic interneurons in the VTA [6,108]. Other inputs to the GABAergic NAc neurons include glutamatergic afferents from the PFC that have been proposed to be very significant in addiction development [108]. Note that drug exposure induces the expression of many markers, one of which is a well-established long-lasting expression of ΔFosB [113,114,115], occurring only in the D1 dopamine receptor-expressing medium-sized interneurons (D1-type medium spiny neurons (MSNs)) [109]. The accumulation of ΔFosB being mainly due to its extraordinary long-life and stability [113,116]. The expression of ΔFosB is associated with increased locomotor sensitivity to cocaine and increased conditioned place preference (CPP) [117,118] and is considered as one marker for repeated exposure to drugs of abuse. ΔFosB is found to be important for the structural and synaptic plasticity after drug exposure [117,119,120] and controls the activity of several other transcriptional and epigenetic regulatory proteins, like the proteins that are important for glutamatergic synaptic function [121,122,123]. Transition to addiction involves neuroplasticity in many limbic regions and is thought to develop through cascades of dysfunction, that beginning with dopamine signalling in the VTA, which subsequently affects target regions including the NAc, dorsal striatum, orbitofrontal cortex, PFC, and amygdala and ultimately facilitates the transition from use to abuse and/or dependence [124].

Dopamine plays an essential role in the motivational aspect of reward [125,126,127]. However, the unitary account of addiction involving dopamine has been challenged by several studies [128]. Indeed, drugs of abuse also sensitise noradrenergic and serotonergic neurons via non-dopaminergic mechanisms [129,130,131,132,133] and rewarding drugs such as opiates or psychostimulants induce different behavioural and neurobiological responses [134]. There are no efficient or approved pharmacological treatments for psychostimulant use disorders (PSUD) [135], although promising agents and approaches have been described for treating various addictions [136,137]. Among them are orexins/hypocretins involved in arousal, stress, anxiety, feeding, appetite and satiety, reward and addictive behaviours [138,139]. Their antagonists modulate reward and drug-associated mesolimbic dopamine signalling and they have been shown to be efficient in experimental animal models using self-administration paradigms to evaluate the reinforcement and the motivation for drugs of abuse [140,141]. Other targets like the metabotropic glutamate receptors [142,143,144,145] and agents like opioid peptides or antibiotics have also been reported as potential treatments against drugs of abuse [146,147,148]. Hopefully, ongoing clinical trials will lead to the necessary treatments required for these important health issues.

Increasing evidence implicates these various mechanisms of gene regulation in the long-lasting changes that drugs of abuse induce in the brain, and offer novel inroads for addiction therapy. This transcriptional and epigenetic model of chronic drug action also provides a plausible mechanism for how environmental influences during development can increase or decrease the risk for addiction later in life [149,150,151]. Drugs alter the transcriptional potential of genes, from the mobilisation or repression of the transcriptional machinery to epigenetics. The drug-induced changes in epigenetic regulation induce highly stable changes in the brain that may mediate the addicted phenotype [6,111,149,152].

Multiple drugs of abuse prompt changes in histone acetylation in the brain [7]. More interestingly, histone acetylation correlates with an increase in the expression of genes induced by drugs of abuse [8,9]. A genome-wide mapping after cocaine exposure at gene promoters showed increased levels of H3 and H4 acetylation in the NAc [153,154,155]. Cocaine increases H4 acetylation on the *fosb* promoter in the striatum [156]. Alcohol withdrawal increases HDAC activity and decreases histone acetylation in the mouse amygdala. Exposure to Δ9-THC, the psychoactive substance in cannabis, also increases HDAC3 activity [157,158,159,160]. Other studies show that HDAC inhibitors potentiate CPP and locomotor responses to psychostimulants [153,154,161]. Further, prolonged inhibition induces changes in the opposite direction [162,163]. Overexpression of HDAC4 or HDAC5 decreases behavioural responses to cocaine, whereas deletion of HDAC5 hypersensitises mice to the effects of drugs [154,161]. SIRT1 plays an important role in addiction and substance abuse [155,164] by binding several histone and nonhistone proteins [165,166], including nuclear factor-κB (NF-κB), several forkhead box (FoxO) proteins, and HATs (e.g., p300/CBP-associated factor). SIRT1is implicated in diverse aspects of drug tolerance and dependence [164,167,168,169]. SIRT1 overexpression increases behavioural sensitisation in the NAc, and its silencing reduces CPP. These epigenetic processes are used in cocaine model studies [167,170]. Furthermore, SIRT1 and SIRT2 induction is mediated via the drug-induced transcription factor ΔFosB. This induction leads to increased H3 acetylation and increased ΔFosB binding at their gene promoters. Therefore, sirtuins are downstream targets of ΔFosB, which is a mediator of the molecular mechanisms induced by drugs of abuse [155,156,164,171,172]. 

On the other hand, histone methylation is directly regulated by drugs of abuse, suggesting an important role in regulating drug-induced behaviours for this epigenetic mechanism as well. Indeed, the specific histone methylation marks, H3K9me2 and H3K27me2, display regulation at numerous gene promoters after chronic cocaine treatment [155,173,174]. Reduced levels of H3K9me2 in the NAc are facilitated by the cocaine-induced downregulation of two HMTs, G9a and G9a-like protein (GLP), which catalyse H3K9me2 formation. This dramatically represses its inducibility by subsequent drug exposure. Remarkably, this cocaine-induced suppression of *G9a* was found to be mediated by ΔFosB by binding and repressing its promoter. This led to a consistent accumulation of ΔFosB after chronic cocaine uptake, suggesting a functional feedback loop between G9a and ΔFosB. Moreover, G9a and ΔFosB share many target genes [2,6,53,69,155,174], including the *Cdk5* and *NFκB* subunit genes [152,174,175,176,177]. Thus, cocaine increases the level of *Cdk5* expression by inducing the binding of ΔFosB to the *Cdk5* gene. This is followed by the recruitment of several proteins and specific chromatin remodelling factors. Ultimately, all these binding cascades reduce the repressive histone methylation of the *Cdk5* gene promoter by the cocaine-induced suppression of G9a [6,154,174,176]. Interestingly, *Cdk5* was found to affect the circadian system indirectly through regulating other proteins that are involved in the clock mechanism (Per2) [178]. In contrast, with chronic amphetamine, ΔFosB was found to bind to its promoter and recruit several HDACs (HDAC1, SIRT1), inducing increased repressive histone methylation at this promoter perhaps related to G9a binding [171,174]. Therefore, these drug-induced histone modifications of specific drug-regulated genes, recruit many additional proteins and ultimately show different regulations signifying either transcriptional activation or repression complexes. HDMs also enhance drug-associated behaviours. The lysine demethylase 6B (KDM6B) is upregulated in the mPFC during cocaine withdrawal and is known to regulate drug-associated reward memory. KDM6B inhibition disrupts both, reconsolidation of cocaine-conditioned memory and reinstatement, suggesting dual effects of KDM6B in cocaine drug-seeking behaviour [179]. In alcohol dependence, the upregulation of KDM6B is associated with epigenetic regulation of signalling pathways by a decrease in the H3K27me3 level, consistent with its known demethylase function [180]. In parallel, KDM5c connects H3K4me3 to drug-elicited behaviours. Its knockdown occurring after consolidation increases global H3K4me3, but inhibits amphetamine-induced CPP [181,182,183]. 

Multiple studies have associated DNA methylation and addiction, yet only a limited number of genes concerned are known, such as methylated CpG-binding protein 2 (MeCP2), cyclin-dependent kinase-like 5 (*Cdkl5*), and protein phosphatase type-1 (PP1) [183,184]. MeCP2 is a prominent MBD reader of DNA methylation, the mutation of which is associated with Rett syndrome, a neurodevelopmental disorder classified as belonging to the autism spectrum disorder in the DSM-IV [107,185]. Mecp2 is well characterised for binding 5-mC, as well as 5-hmC [7,186]. Its activity is regulated through both phosphorylation and expression mechanisms by cocaine and anti-depressive agents that are necessary to reinterpret DNA methylation acquired during early development [130,187]. MeCP2 expression is necessary to convey the effects of the psychostimulants, in particular those of cocaine [188]. It is best known for its role in repressing transcription by recruiting a member of the class I HDACs and several other transcriptional repressor complexes, but it can also associate with DNMT1, with histone methyltransferases, or with histone acetyltransferases [7,189,190,191]. The level of MeCP2 protein present at a given time is of vital importance and can be modified by several compounds, including drugs of abuse [188]. Note that *Cdkl5* was the first direct MeCP2 target gene shown to be repressed by DNA methylation in response to cocaine through an in vivo direct interaction with Mecp2 in the adult brain [184]. Interestingly Cdkl5 was also reported to belong to the same molecular pathway of MeCP2 and responsible for the early-onset seizure variant of Rett syndrome [192]. Aside from Mecp2, involved in learning and memory processes [193], protein phosphatase type-1 (*PP1*) initially reported as a memory suppressor gene [194], was found to be regulated by covalent DNA methylation involved in memory formation during fear conditioning experiments [195]. Thereafter, it was shown to be repressed by cocaine through a MecP2 binding-mediated mechanism in cocaine-induced behavioural sensitisation [186], and in passive or voluntary drug administration [196,197]. Therefore the role of *Mecp2*, *Cdkl5,* and *PP1* genes highlight alterations of cognitive function by cocaine. This is consistent with the hypothesis that addiction can be considered as a form of memory in which normal learning and memory processes are hijacked by exposure to drugs of abuse with robust and long-lasting addiction-related memories [198]. Cocaine-induced *Cdkl5* gene hypermethylation correlates with the increased DNMT3A and DNMT3B expression induced by cocaine [7,130,186,196]. On the other hand, TET1 was downregulated following cocaine administration [199]. Consistent with cocaine-induced DNA methylation changes, *Dnmt* and *Tet* genes are modulated by cocaine in various conditions in brain structures [196,200,201,202], and in a time-dependent manner (submitted article [203]). Despite, in genome-wide studies, very few modifications in global brain DNA methylation upon cocaine exposure were detected [199,204,205,206,207,208]. The expression of MeCP2 in the dopaminergic projection areas was increased with cocaine [130,188]. Knockdown of MeCP2 decreases drug intake and reduces drug-behavioural responses like CPP [2,130,188,190,209]. Acute or chronic cocaine treatment increases the *fosB* mRNA expression, which co-occurs with a decreased methylation at the *fosB* promoter in the NAc. This is consistent with the stable increase in ΔFosB protein expression with cocaine exposure. Even more interesting is the role of *fosb* in affecting the circadian system indirectly through controlling the activity of several regulatory proteins [9,186,210,211,212]. DNMT3a was explicitly associated with CpG methylation and addiction [202]. Acute or chronic cocaine increased the expression of Dnmt3a in the NAc, causing DNA hypermethylation and increased MeCP2 binding at the PP1Cβ promoter, decreasing the psychostimulant reward [186,196,197,201]. On the opposite, reduction of Dnmt3a activity using either knockout mice or an inhibitor injected directly into the adult NAc, increased the behavioural responses to cocaine [201]. Besides, TET enzyme expression, involved in the demethylation process, was decreased in the NAc following cocaine treatment. This was associated with an increase in 5-hmC expression at a specific gene locus [26,199]. Although the long-lasting suppressive consequences of CpG methylation on gene expression are well known, particularly for genes expressed in a cell or tissue-specific manner, it is still unclear to which extent the alterations in DNA methylation are rhythmic. Furthermore, global changes in DNA methylation have been described in an addiction model [208], however, methylation at the CCG gene promoters is still under examination in this model.

Several studies have provided evidence for the involvement of miRs in addiction behaviour, as they were found to influence the expression of many proteins engaged in addiction [30,213]. Those miRs are targets for genes involved in synaptic plasticity and drug addiction [214,215]. MiR-134 is brain-specific, regulated by SIRT1, and involved in the regulation of CREB and BDNF levels. Both these proteins are importantly involved in many neuronal functions and are regulated by cocaine as well. Additionally, several lncRNAs exhibit expression changes in the NAc induced by heroin, suggesting a novel target for regulating addiction-related behaviour and gene alterations [216]. Cocaine induces the expression of the CREB-dependent miR-212 in the striatum, which decreases the rewarding effects of cocaine by interacting homeostatically with MeCP2 to regulate BDNF expression and thus cocaine intake [6,30,188]. miR-212 and miR-132, actually, belong to a polycistronic cluster, and it was proposed that Mecp2 mediates cocaine and food effects by modulating the processing of this polycistronic pri-miR-212/132 cluster into pre- and mature forms [202], in agreement with studies having reported that Mecp2 regulates RNA splicing [217] and suppresses nuclear pri-miR processing by regulating the DGCR8/Drosha complex [218]. Similarly, miR-124 and miR-181a, being regulated in the brain by chronic cocaine treatment, operate through the CREB-BDNF complex [219,220]. They also play a role in the expression of the dopamine transporter [221]. In addition to cocaine, miR-190 is downregulated by opioid receptor activation [222]. The let7-d family is upregulated by chronic morphine exposure. MiR-9 is upregulated with chronic alcohol treatment and targets the dopamine D2 receptor [223], providing further examples of the effect of drugs of abuse on miRs expression.

## 6. Clock Genes and Addiction

The 24 h light–dark cycle, which characterises the Earth’s daily rhythms, harmonises many behavioural and physiological rhythms, including the sleep-wake cycle and crucial cognitive/neuronal functions, some of which are involved in neuropsychiatric disorders. This daily periodicity is disturbed by drugs of abuse, which provoke disturbances in sleep, mood patterns, and other behaviours [35,41,224]. For example, drugs of abuse not only disrupt sleep architecture but also its timing by acting as an environmental cue that phase-shifts circadian rhythms [35,225]. Moreover, biological and behavioural rhythms may respond with different phase shifts to drug use, which may result in mood disorders and lifestyle disruptions, explaining the co-morbidity with drug consumption [226,227,228,229]. For example, alcohol acts on cholinergic and adenosine neurons, which are mediators of the sleep system. The alcohol-induced sleep disturbances may also result in a reset of the circadian clock phase. Although acute alcohol intake can aid in sleeping as a somnolent, in people with alcohol use disorder (AUD), alcohol intake results in interrupted sleep routines and altered eating and activity patterns. These adverse effects are much more potent and may endure during withdrawal and thereby cause relapse [35,230,231].

In addition, disruption of sleep and circadian rhythmicity is a common symptom of many psychiatric disorders, including drug dependence [35,232,233]. In parallel, individuals with a compromised circadian clock, or with circadian-based mood disorders, like depression, are more prone to drug dependence [234,235,236]. The substantial interaction between the circadian timing system and other molecular systems suggests that the risk for substance use disorders (SUDs) might be affected by genetic disturbances of the molecular clock mechanism [35,232]. Indeed, clock disruptions result in detrimental effects of behavioural responses to drugs of abuse and an increased propensity to self-administer them [237,238,239].

Clock genes are involved in both the behavioural response and the enticement motivation for drugs, thereby playing a major role in the regulation of drug reward and reinforcement. Disrupted clock protein function may cause an increase in cocaine liability and decreased CLOCK function increases vulnerability for cocaine use and its rewarding effects as assessed in the CPP paradigm [233,240]. Similarly, pioneering studies in Drosophila showed that the *Clock* gene modulates acute ethanol sensitivity. Moreover, flies lacking functional *clock*, *cycle*, and *period* genes failed to behaviourally sensitise to cocaine, despite repeated exposure [241,242]. Remarkably, Clock, which is highly expressed in striatal regions of the mammalian brain, has been associated with the development of emotional memory, sleep, and food entrainment [243,244,245]. In addition to Clock, Bmal1 has been reported to be involved in modulating drug reward, with one of the transcriptional targets of the BMAL1 protein being the monoamine oxidase A (*Mao-a*) gene, which encodes a DA metabolising enzyme [239,246]. Additionally, Bmal1 plays a vigorous role in circadian clock-controlled xenobiotic metabolism, meaning that drug detoxification is clearly affected by the time of the day [247]. Moreover, the *Mao-a* gene is also a transcriptional target of PER2 proteins [246]. *Per2* positively regulates *Mao-a* expression. Consequently, mice with a *Per2* mutation *(Per2^Brdm1^)* showed a decrease in *Mao-a* expression in the NAc and VTA, resulting in increased midbrain DA levels and subsequently an increase in cocaine sensitivity [239,246]. These results indicate that the transcriptional activation of *Mao-a* may depend on a functional *Per2* gene.

The PER repressors of the circadian clock are known to be linked to addictive behaviours as well. Initially, *mPer1* and *mPer2* mutants showed no difference in sensitivity to acute cocaine administration. However, after repeated injections, a loss of the cocaine behavioural response and thus a complete lack of cocaine reward became manifest in *Per1* mutant mice, whereas *Per2* mutants emerged to be hypersensitive with a strong cocaine-induced CPP [239,248]. In addition, methamphetamine treatment changed the striatal expression of the Per genes in a way similar to the alterations in the rhythms of locomotor activity [234,249]. mPer1 controls morphine dependence through its effects on extracellular signal-regulated kinase (ERK) signalling. Targeted disruption of mPer1 by a DNA-based enzyme (DNAzyme), prevents the increased ERK expression that normally is induced with morphine treatment. Moreover, mutant mice support a role for Per2 expression, although not Per1, in mediating the modulatory time-of-day effects on the response to ethanol [248]. mPer2 was found to influence alcohol consumption [234,240,250] and mice with a mutation in the PAS domain of Per2 voluntarily consumed more ethanol and displayed an advanced onset of nocturnal drinking. These findings indicate a relationship between alcohol intake and the *Per2* gene, which might be functionally relevant also for addiction in humans [251,252]. The above data clearly show the participation of the core clock system in the modulation of drug-related behaviours and drug-induced plasticity in animal models. 

Another line of evidence emphasising a close relationship between the circadian system and the addiction-driven molecular network can be found in the dopaminergic system [35,232]. Within the mesolimbic nuclei, nearly all aspects of dopaminergic activity including neuronal firing patterns, neurotransmitter synthesis, release, degradation, and post-synaptic actions display diurnal variation through Clock and Bmal1 transcriptional targets. This diurnal variation may underlie the variations observed in the behavioural responses to drugs [233,246,253,254,255]. In particular, this concerns the part of the dopaminergic system that involves the DA neurons in the VTA projecting to target regions including NAc and PFC [35,232]. Within the VTA, the expression of DA receptors was found to be rhythmic, as well as that of tyrosine hydroxylase (TH) and MAO-A, two enzymes responsible for the synthesis and degradation of DA, respectively [233,246,253,254,255,256]. At the presynaptic DA release site of VTA neurons projecting onto the postsynaptic GABAergic medium spinal neurons (MSNs) in the NAc, a variety of mechanisms are under circadian transcriptional control by direct binding of CLOCK to DA-related genes. Pre-synaptically, CLOCK negatively regulates the transcription of TH to affect DA synthesis. CLOCK also positively regulates the activity of the neuropeptide cholecystokinin (CCK), which negatively influences DA output and MAO-A activity. CLOCK and Bmal1 differentially regulate DA transmission, as Bmal1 specifically regulates the expression of Drd3. In parallel, functional studies support the role of Clock in the VTA and Bmal1 in the striatum as respectively negative and positive regulators or drug reward. These regulatory genes may be considered CCGs, as they contain canonical E-box sites on their promoters that are bound by CLOCK and Bmal1 [246,255,257]. Therefore, the circadian timing system directly intermingles with the VTA–NAc projection that is involved in reward-related behaviour [35,232]. 

Furthermore, an altered glutamatergic system was found to contribute to the drug-seeking phenotype, and drug-induced plasticity [258]. Extracellular levels of glutamate and GABA in the striatum both present a daily rhythm that peaks at night [259], but their normal daytime decrease was prevented by melatonin, suggesting that melatonin modulates those striatal rhythms in glutamate and GABA transmission [260]. Glutamate is co-released from dopaminergic VTA projections [261]. Melatonin release from the pineal gland is considered a major hormonal output of the master circadian clock. Melatonin, a molecule that is also receiving attention for SUD, is exclusively released at night and re-adjusts sleep and other rhythmic physiological events [262,263]. In response to pinealectomy, daily *Per1* rhythms were abrogated in striatal regions, but not in other regions of the brain [264]. Nevertheless, note that there are no proven straightforward projections from the SCN to the striatal regions. On the other hand, melatonin receptors were found to be differentially regulated in the striatum following chronic cocaine treatment, whilst circulating melatonin levels were found to remain unaffected by this treatment [265]. Thus, the daily rhythm in melatonin release could be important for synchronising these striatal regions. Adjustments in melatonin signalling in striatal regions might affect mood, motivation, or other processes related to addiction (reward processing, behavioural responses), but as yet its position in mediating drug-induced responses is unclear. 

Among the neuropeptides that are regulated by circadian rhythms, neuropeptide Y (NPY) and orexin share common functions, notably in the regulation of appetite and satiety. Moreover, both are under the influence of drugs of abuse and natural reinforcers such as food or sugar. NPY is widely expressed throughout the central nervous system and often is co-secreted with GABA or glutamate [266] and plays a crucial role in cortical excitability, stress response, food intake, and circadian rhythms [267]. The *orexin* gene has a more restricted expression pattern being only transcribed in the lateral hypothalamus (LH). Orexinergic neurons regulate metabolism, feeding, and reward controlling physiological and hedonic appetite [268]. The *orexin receptor-1* gene [202] regulates the reinforcing and rewarding properties of cocaine [141], while *orexin* gene expression is modulated by cocaine [202]. In addition, the *orexin* promoter was reported to be modulated by drugs of abuse through DNA methylation, as well as its receptor genes [202,269,270,271,272]. Furthermore, note that orexins and NPY antagonists have been characterised as promising therapeutic targets for addictive behaviours, drug abuse disorders, or metabolic diseases [138,140,266,267,273,274], demonstrating a prominent link between rewarding agents, potential therapeutic tools, and epigenetic mechanisms.

Overall, these data show that circadian networks overlap with various addiction-related signalling pathways involving genes implicated in physiological and cognitive functions, some of them being regulated by DNA methylation. It will be of critical importance to determine the afferent signalling pathways involved, in order to understand how these changes, modify responses to drugs of abuse and addictive behaviour. Recently, an even more complicated scenario evolved concerning the interaction between the circadian system and drugs of abuse, as the latter may not only disrupt circadian rhythms, but desynchronised circadian cycles may also influence addictive behaviour and serve as a risk factor for SUD [226,275]. Therefore, such a bidirectional mechanism would create a down-ward vicious circle. 

Several hyper-hedonic behaviours were found in ClockΔ19 mice, i.e., amplified locomotor responses, abridged depression- and anxiety-like behaviour, increased sensitivity for self-stimulation, and increased dopaminergic cell activity in the VTA [39]. Other clues suggest that disturbances of the circadian timing system may result in an increased vulnerability to addiction and related behaviours [35]. ClockΔ19 mutants showed increased cocaine-induced CPP as well as elevated ethanol consumption [35,39]. ClockΔ19 mice also display augmented DA synthesis and activity primarily during the light cycle, explaining the increased daytime sensitivity to the reinforcing efficacy of cocaine observed in these mice (26). 

Without a central clock, animals seem to be more sensitive to drugs of abuse (22), as methamphetamine robustly re-established activity rhythms in SCN-lesioned animals [225,234,249,276]. Excision of the central SCN clock also results in expression discrepancies in the reward circuitry of the rodent brain suggesting a function of SCN in the diurnal regulation in this circuitry [109,232,255,277,278]. Moreover, in humans, shift work appears to precipitate an increased risk of intense alcohol consumption and relapse [226,227,228,230]. The expression levels of dopamine transporter and TH were different in SCN-lesioned animals compared to SCN-intact ones [255]. Furthermore, SCN-lesioned animals showed no diurnal pattern in locomotor activity, but an enhanced acquisition of CPP [257]. 

Furthermore, in ClockΔ19 mice, glial glutamate uptake and glutamate transporter (GLAST) levels are significantly reduced, increasing glutamatergic tone and critically decreasing *Glast* expression in the VTA [258]. Moreover, expression of the vesicular glutamate transporter 1 (VGlut1) protein in synaptic vesicles prepared from the whole brain revealed a diurnal rhythm, which was lost in mice lacking Per2, indicating that circadian clock components also amend glutamatergic vesicular sorting [279]. The same mice also showed downregulated levels of the glutamate receptor subunit GluR1 and glutamate transporter Eaat1, resulting in atypically high levels of striatal glutamate associated with an increase in alcohol intake. Therefore, data from ClockΔ19 and Per2 mutant mice implicate an important role for these genes in regulating the expression of key genes involved in glutamatergic signalling in the striatum [251]. Together, the augmented dopaminergic and glutamatergic systems may underly the molecular mechanisms triggering the increased tendency for addiction-like behaviours when the circadian timing system is disturbed.

## 7. Epigenetic Connection between Circadian Rhythms and Addiction

Several histone-modifying enzymes that are regulated by the circadian clock have been linked to functional abnormalities found in addiction models. For instance, sirtuins are associated with behavioural adaptations that enhance the rewarding effects of drugs, as well as with the circadian system by interacting with the clock genes. Many studies have linked circadian rhythms with the risk of neuropsychiatric disorders, while others investigated the regulatory role of epigenetic factors in circadian rhythms. Yet only a few studies addressed whether epigenetic regulation in the circadian system may be linked to neuropsychiatric disorders. Prader-Willi syndrome (PWS) is the first neuropsychiatric genetic disorder with evidence of disrupted circadian epigenomics. PWS results from a chromosome deletion, considered to be caused by the loss of a lncRNA gene, 116HG. A study in mice lacking 116HG showed altered expression of several clock genes and energy use in the brain [280,281,282]. Another pilot study connecting epigenetics, clock genes, and psychiatric disorders concerns miR. The precursor of miR-182 was found to carry a single nucleotide polymorphism (SNP) associated with late insomnia. CLK is a predicted target of miR-182 and the regulatory relationship was validated. This relationship between this SNP, the expression of miR-182, and its circadian target warrants further investigation [283].

The enormous genomic reprogramming that occurs in addiction has inspired investigations on the epigenetic changes occurring in the brain. A promising view is that key chromatin remodelling from histone modifications to DNA methylation plays a crucial role in the regulation and stability of drug-mediated neuronal gene programming, leading to addictive behaviours [2,173,284]. As genetic studies had limited success in finding proof to link circadian rhythms to addiction this attracted attention to the possibility that circadian epigenetic factors are involved in addiction. We know by now that the adaptable and dynamic circadian timing system is regulated by the interaction between environmental cues and the molecular clock. As described earlier, cues like light, food, temperature, stress, hormones, and drugs, shape the circadian phenotype by regulating epigenetic factors including non-coding RNAs [60,72]. 

**Figure 1:**
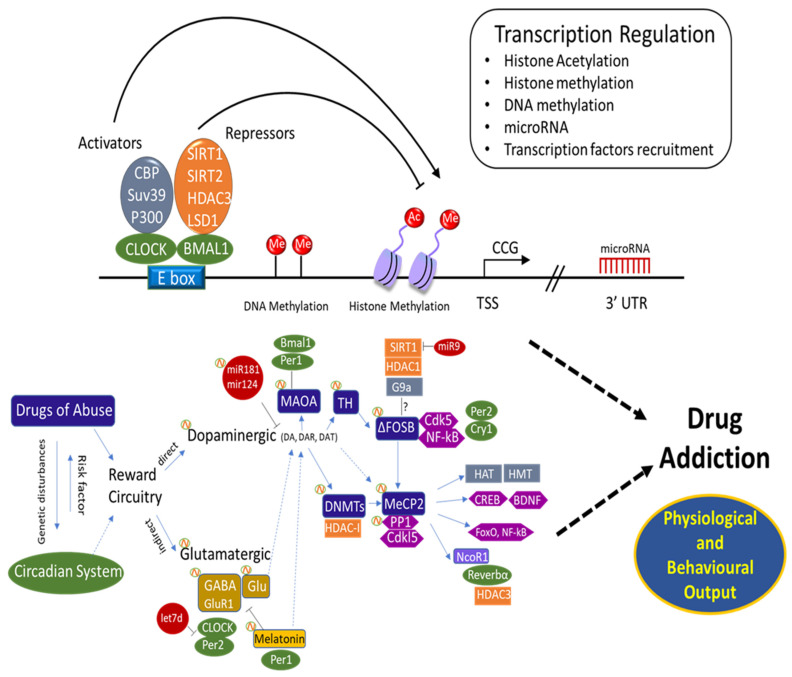
Circadian epigenome and reward circuitry in addiction. A schematic representation of the core clock machinery is shown. The activating heterodimer CLOCK:BMAL1 binds to the E-box elements on the genome, controlling a large number of genes. CLOCK:BMAL1 action is counteracted by the PER and CRY repressor proteins. Additional regulators and chromatin remodelers contribute to circadian gene expression. Among the genes controlled by the clock, a number of them are key reward system regulators. The molecular clock has also been shown to interplay with several transcription activators and repressors complex genes.

Considering that cocaine modulates the expression of some circadian genes, as well as the *Dnmt* and *Tet* genes with opposite effects on global DNA methylation, one can speculate on the existence of an epigenetic “connection” between drug addiction and the circadian timing system. Moreover, the levels of MeCP2, induced by cocaine and affecting the overall structure of brain chromatin, fluctuate with a circadian period, ultimately resulting in a circadian-dependent regulation of *MeCP2* target genes [285]. In the same vein, PP1 is also considered a post-translational regulator of the mammalian circadian clock [285,286]. Interestingly in addition to the involvement of PP1 in the response to drugs of abuse as described above, PP1 also regulates both the stability of the circadian protein PER2 [287] and post-translational regulation of the circadian clock [286,288]. Thus, PP1 well illustrates a connection between circadian rhythms and drugs of abuse [289]. Other studies showed that MECP2 binds to the nuclear receptor corepressor (N-CoR) complex in the brain [189]. N-CoR has a critical role in developmental processes as well as in circadian rhythms [290]. It binds and activates HDAC3, resulting in altered regulation of clock genes and circadian behaviour [63]. In addition, the NCOR1-HDAC3 complex regulates the circadian expression of the core clock gene BMAL1, which mediates the repression of *Reverb-alpha* [290]. This involvement in circadian regulation demonstrates that BMAL1 repression is facilitated by the Rev-erbα/NCoR1 complex [291,292]. Furthermore, FosB is induced in the SCN by light pulses in the (subjective) night. On the other hand, FosB also appeared to be constitutively expressed at high levels in the SCN throughout the LD cycle. However, further immunohistochemical analysis suggested that ΔFosB was the protein product accounting for this constitutive expression [156,211,212]. Thus, ΔFosB is not only linked directly to several addiction-related behaviours [293,294,295], but may also play an indirect role by affecting the circadian system. Moreover, miR-132 represents a direct link between light and chromatin remodelling: it is induced by photic entrainment cues via the (MAPK)–CREB signalling pathway [30,296,297]. Incorporation of this pathway suggests an involvement in the development of addiction behaviour [68,95,101,298]. Although the aspects described and the common signalling cascades involved in the two systems underline the existence of a possible connection, still more focused and correlated studies are required to provide solid evidence (Figure 1).

In a recent study, we found that cocaine and sucrose differentially regulate genes involved in DNA methylation and circadian gene expression in certain rat brain structures (PFC, CPu) (submitted article [203]). A global DNA methylation analysis showed an increase in 5-mC by cocaine, consistent with repression of critical core-clock genes by cocaine. The DNA methylation state of some core-clock genes (*clock*, *cry2*) was consistent with an increase in global DNA methylation in response to cocaine (submitted article [203]). Yet, additional gene-specific DNA methylation experiments are required to ascertain a definite link between the circadian timing system and its contribution to addiction.

Therefore, while DNA methylation was initially considered as a more simple mechanism than the histone code associated with tremendous post-translation modifications [299], many issues remain to be addressed in this part of neuro-epigenetics. On one hand genome-wide studies have identified many DMRs, each one having a number of potentially CpG target sites for methylation or demethylation. However global DNA methylation analyses have reported no or little changes in response to drugs of abuse [199,204,205,206,207,208], suggesting that it is rather the distribution and the position of CpG target sites that may contribute to drug abuse-related phenotypes. On the other hand, the functional role of DNA methylation in gene expression remains an important and challenging issue to address. While many genes involved in functions such as circadian rhythms, learning and memory processes, arousal and appetite or satiety are modulated by drugs of abuse and natural reinforcers, some of them, to some extent, are also Dnmts and/or Tets targets [194,195,196,202,203,300,301]. It therefore appears reasonable to hypothesise that some are regulated by DNA methylation, whereas others can be independent of it, notably through one-carbon metabolism.

As there is still not much known about this interaction, further investigations are needed, using for instance pharmacological or gene silencing approaches. However, it is important to keep in mind that all these epigenetic factors, DNA methyltransferases, histone-modifying enzymes, such as HDACs, HMTs, and histone demethylases, are known to interact. Their mutual recruitment results in a high complexity of the epigenetic control of addiction and circadian rhythms.

## 8. Future Perspective

The accumulating evidence discussed here proposes a promising opportunity for a better understanding of the neuronal mechanisms underlying drug addiction, by integrating the knowledge on the circadian timing system. Unfortunately, many aspects of this interaction are still poorly understood, in particular its regulatory epigenetic aspects. In addition to the discovery of clock genes and clock-controlled genes associated with the circadian timing system, there is a need to explore how epigenetics is integrated within the molecular clock system. Although circadian rhythms are considered a major phenotype to study in neuropsychiatric disorders and epigenetic changes in addiction are well-known, circadian epigenomics within the perspective of neuropsychiatric disorders has not received a lot of attention yet. Nevertheless, describing such networks together with their regulations may aid in understanding the link between circadian timing and these disorders and thus in understanding human behaviour.

Given the complexity of the system, most studies in this emerging research field have been performed in mouse models and only a few in humans. In humans, as is the case in animals, studies provide non-reliable information when the time of day is not respected and/or considered. Furthermore, genomic and epigenomic studies should take diurnal variations into account, i.e., variable gene expressions or epigenetic markers depending on the time of day. Although animal models provide alternatives that can overcome many of the constraints found in human brain research, they still have their own limitations: findings in animal rodents on epigenetic regulation may not translate directly to humans. In addition, the experimental/technical biases are also to be considered. The optimal use of the different models may lead to a better comprehensive insight into the regulatory network and its significance to the human circadian-related disorder, drug addiction.

As circadian genes modulate drug-induced pathologies, potential therapeutic agents (drug targets) may be established using the findings from genetic and epigenetic studies of the circadian timing system. Drug targets might be hidden in the list of individual components of rhythmic expression or epigenetic control of these rhythms, that need further investigation. In theory, new therapies using drugs to modify the epigenome might be much easier than correcting mutated genes. Therefore, epigenetic drugs may have great potential in treating neuropsychiatric disorders. Innovative approaches are hastening advances in understanding the epigenetic state of the individual at gene promoters and the whole genome to conquer the caveats. Nevertheless, more genome-wide association studies have to be applied to discover further molecules, functionally meaningful chromatin codes, and functional pathways.

Investigations of epigenetic mechanisms have proven profitable in various fields, including the fields of drug addiction and chronobiology. Taken together, it is clear that epigenetic mechanisms are critically involved in both addiction and the regulation of the circadian clock, which raises the question of whether this may represent a link between drug addiction affected by a derailed circadian clock. Studying the environmental impact and gene-environment interactions, using circadian-related phenotypes and biomarkers, opens multiple avenues that require further exploration and could result in potential therapeutic intervention. As the circadian cycle is an extremely environment-dependent system, this is one of the best approaches to link environmental conditions with drug addiction and epigenetics. A better understanding of epigenetic mechanisms in animal models that mimic human conditions should help to usher in a new area of drug development against addiction.

## Data Availability

Not applicable.
